# Lifetime commercial heterosexual behavior among HIV negative elderly men from rural Chengdu, China: a modified knowledge-attitude-practice perspective

**DOI:** 10.1186/s12889-021-12139-z

**Published:** 2021-11-15

**Authors:** Yi Yang, Hua Deng, Huan He, Shuang Feng Fan, Yuan Li, Xia Wu, Na Li, Jing Xi, Jing Xu, Jie Xiao, Rui Ping Liao, Wei Xiao

**Affiliations:** 1 Department of Social Medicine and Health Administration, School of Administration, Chengdu University of Traditional Chinese Medicine, 1166 Liutai Avenue, Wenjiang District, Chengdu, 611137 China; 2 Department of Urology, Chengdu Second Peoples’ Hospital, 10 Qingyunnan Road, Chengdu, 610017 China; 3 School of Public Administration, Southwestern University of Finance and Economics, 555 Liutai Avenue, Wenjiang District, Chengdu, China; 4 Department of HIV/AIDS prevention, Chengdu Center for Disease Control and Prevention, No.4, Longxiang Road, Wuhou District, Chengdu, 610000 China; 5 Department of Epidemiology and Health Statistics, School of Public Health, Chengdu University of Traditional Chinese Medicine, 1166 Liutai Avenue, Wenjiang District, Chengdu, 611137 China; 6 Department of HIV/AIDS prevention, Pengzhou Center for Disease Control and Prevention, 331 Longta Road, Pengzhou District, Chengdu, 610000 China

**Keywords:** Sexual behavior, Elderly men, Knowledge-attitude-practice (KAP) model, HIV, China

## Abstract

**Background:**

China is facing big challenges to achieve the “90–90-90 targets”. The HIV prevalence of elderly (≥50 years) men have been steadily increasing in China, mainly through the sexual transmission route, but sexual behaviors of them are far from well-studied. In 2019, elderly men accounted for 59.2% of HIV/AIDS cases in Sichuan, China.

**Methods:**

The research design is a cross-sectional study. Face-to-face interviews were conducted among 795 HIV negative elderly men from rural Chengdu, capital City of Sichuan. Bivariate and multivariate logistic regression models were applied to examine factors associated with commercial heterosexual behavior from a modified Knowledge-Attitude-Practice (KAP) perspective.

**Results:**

129 (16.23%) respondents admitted high-risk sexual behaviors, including 11.07% commercial heterosexual behavior, 6.16% extramarital, 2.89% casual and 0.25% homosexual behavior, and no one used condom consistently. 427(68.43%) had ever gotten HIV-related Health Education (HRHE), mainly through mass media (70.49%). The HIV-related knowledge awareness rate was only 31.41%. Migration history (AOR =2.46,95% CI = 1.02–5.91), age(≥60 vs. 50–59, OR = 0.41, 95% CI = 0.19–0.91), receiving HRHE from mass media (OR = 0.37, 95%CI = 0.16–0.85), marital status (married vs. never married, OR = 0.04, 95%CI = 0–0.52), and undecided (AOR =0.02, 95%CI = 0.01–0.09) and objection (AOR =0.04, 95%CI = 0.01–0.1) attitude toward commercial sex were related to lifetime commercial heterosexual behavior.

**Conclusions:**

High-risk sexual behaviors are common among elderly men from rural areas in Chengdu. Receiving HRHE from mass media and undecided and objection attitude toward commercial sex prevent elderly from being involving in commercial heterosexual behavior. According to the results, health facilities should continue to conduct systematic interventions, paying more attention to 50–59 years old group. Sex and condom use need to be talked in public. Working with mass media, health facilities give elderly men education not only focusing on HIV/AIDS, but also on knowledge and skills of condom use.

## Introduction

Benefiting from a scale-up of antiretroviral therapy (ART) and prevention of mother-to-child transmission in the past two decades, annual incidences and mortality rates of human immunodeficiency virus/Acquired Immune Deficiency Syndrome (HIV/AIDS) have been declining [1, 2]. The declining leads to ambitious calls to end the HIV/AIDS epidemic as a public health threat by 2030 [3]. The Joint United Nations Programme on HIV/AIDS (UNAIDS) proposed the “90–90-90 targets”, which aims to have 90% of people living with HIV (PLHIV) knowing their status, 90% of those diagnosed receiving sustained ART, and 90% of those receiving ART achieving viral suppression by 2020 [4].

China is facing big challenges to achieve the “90–90-90 targets” [5, 6]. One of the biggest challenges is that the HIV prevalence of elderly (≥50 years old) men has been steadily increasing, mainly through the sexual transmission route [7–12]. Due to immune functional decline and increased vulnerability to infections [13], HIV infected elderly men quickly progress into AIDS even deaths [9, 13]. Being considered as sexually inactive [14], elderly men had been excluded from the priority of HIV/AIDS prevention programs [15–18], and are not recognized and listed as a key HIV prevention group until 2017 for the first time in China [19]. On one hand, current elderly men are from 20 to 50-year-old group, who are previously sexually active and might be HIV infected as clients of female commercial sex workers (FCSW) [20–25] and men who have sex with men (MSM) [26–30], especially when they migrate [31–33] but not detected. On the other hand, they could be a newly infected group. Compared with other age groups, elderly men have minimal knowledge about HIV [34], condom use among them is even lower [25, 35, 36].

Located in the southwest of China, Sichuan is one of the few provinces heavily affected by the HIV/AIDS epidemic where heterosexual transmission increased from 65.3% of total cases in 2008 to 98.2% in 2019 [37]. People over 50 years old in Sichuan accounted for less than 5% of HIV/AIDS cases in 2005, but over 40% in 2017, and 59.2% in 2019, meanwhile the proportion of elderly men in the population is less than 40% [5, 37, 38] .One of fifth PLHIV in Sichuan are from Chengdu [5, 37], the capital city. Based on case report system at population level [9, 10, 37], heterosexual transmission has been widely accepted as the major route for HIV epidemics in China. Why are elderly men involved in high risky sexual behaviors? Since the 1980s, rural-to-urban migration have been part of their lives for men from rural areas in China, and increased the possibility of being involved in high risky sexual behaviors [31–33]. According to knowledge-attitude-practice (KAP) model [25, 34–36, 39, 40] which has been applied in HIV prevention since 1990s, the hypothesis flows are the following: elderly men are involved in high risky sexual behaviors because they don’t believe these behaviors are highly risky. Why don’t they believe? Because they don’t have enough knowledge. In order to help them to avoid high risky sexual behaviors, it is necessary to give them health education to improve knowledge level. Though KAP model has been proved not as effectively as expected [25], due to their minimal knowledge about HIV [34], current comprehensive HIV prevention strategies in Sichuan is based on KAP [5] .

Sexual behaviors of elderly men in China are far from well-studied. In 2019, 1 year before 2020 when the “90–90-90 targets” are expected to be achieved, our team conducted a cross-sectional investigation to understand sexual behaviors among elderly men in Chengdu, China where HIV prevalence of them have been increasing in recent years and HIV prevalence of MSM has been consistently high [5].

## Methods

### Study site

With the coverage of sentinel surveillance improving [5] and huge number of residents, rural County A in Chengdu ranked one of the top five counties in the number of PLHIV, mainly from male group.

### Study process

The research design is a cross-sectional study. According to the surveillance information from the web-based National HIV/AIDS Comprehensive Response Information Management System (CRIMS), public health workers from County A center for disease prevention and control (CDC) selected the top six towns in HIV infection number to be the study sites. We conducted two pilot investigations to test the questionnaire and to adjust the investigation procedure and manual before the formal investigation.

Male interviewers were trained through three steps: before pilot investigation, pilot investigation and at site. Six male interviewers, who were well trained and pasted the interviewers’ test, conducted the investigation.

### Participants and investigation

Multistage cluster random sampling and multistage probability proportional stratified systematic sampling were applied to recruit eligible respondents. The hierarchy in County A is county-town-village-village group. In four of six towns, multistage cluster random sampling was applied. Firstly, one village from each town was randomly selected, then one village group was randomly selected. All eligible elderly men in the village group were invited to participate the study. In the other two of six towns, multistage probability proportional stratified systematic sampling were applied. According to the total number of elderly men in each village group and their proportion in each town, the sampling size for each village group were confirmed.

Resident roster was used to sample the potential respondents.

The selection criteria included: (1) ≥50 years old; (2) male; (3) live in the current address for at least 6 months. After eligibility was determined and a written informed consent was completed, a 30-min face-to-face structured interview was conducted in a private room by one well-trained male interviewer to make sure the respondents felt comfortable to talk about their sexual behaviors. Local slangs were used to refer sexual behaviors. Completed questionnaires were inspected at the sites to ensure no missing items, wrong information or logical errors. All participants got a big bottle of dishwashing liquid (approximately valued 2 USD) for their participation (no matter completing the questionnaire or not). From June to July 2019, 802 men were recruited, and 797(99.38%) questionnaires were reliable.

### HIV rapid testing

According to the sentinel surveillance routine procedure in the township health centers, HIV rapid testing was conducted through two ways. The first method was sampling a 3-5 ml cubital venous blood of each respondent at the investigation sites and sending it to a local laboratory. An enzyme-linked immunosorbent assay (ELISA) was used for HIV antibody test. The other method was using Dot immunocolloid gold rapid test (Yingke New Technology Co., Ltd.) at the investigation sites where immediate results will be showed. In total, 2 of 797 were fast tested HIV positive.

### Measures

Based on the current HIV/AIDS programs strategies in Sichuan [5] and literature [25, 35, 36, 39, 40], a modified KAP model was applied. Attitudes were reflected by sexual attitude, self-identified vulnerability, and HIV-related sigma. High-risk sexual behaviors included commercial heterosexual behavior, extramarital sexual behavior, casual sexual behavior, and homosexual behavior. Demographic characteristics, migration experiences [31–33], HIV-related health education (HRHE), sexual identity, and sexual desire change were taken as controlling factors for KAP model.

#### HIV-related knowledge

According to United Nations General Assembly Special Session on HIV/AIDS (UNGASS) indicator [40], eight survey questions (China National Eight) (Cronbach’s Alpha = 0.77) were used to assess HIV-related knowledge (detail in Table 3). Respondents received one point for each correctly answered question. The sum of the eight questions represented respondents’ HIV related knowledge level. Respondents were categorized into two groups: ‘know’ (score greater than 5) and ‘do not know’ (score equal and less than 5).

#### Sexual attitude

“How do you think about visiting FCSW?” was asked to measure their attitude toward commercial heterosexual behavior. The response options were: (1) it is understandable as a common human behavior; (2) undecided;(3) could not accept; (4) strongly objection. Besides the above four response options, “admiring” was considered as one option toward “How do you think about extramarital sexual behavior”. The response options toward “How do you think about homosexual behavior” were: (1) it is understandable and he has homosexual behavior; (2) it is understandable but not acceptable; (3) could not accept; (4) could not accept but be involved in; (5) strongly objection; (6) do not know what homosexual behavior is.

#### Data analysis

Seven hundred ninety-five of 797 (99.75%) respondents who were HIV negative were included in the current analyses. Frequencies of nominal variables, mean and standard deviation for interval variables were assessed. Commercial heterosexual behavior was the most common high-risk sexual behavior. T test, ANOVA and chi-square tests were used to examine the relationships between commercial heterosexual behavior and independent variables including HIV-related knowledge, attitude and controlling factors. Binary logistic regression with backward stepwise selection was applied to examine factors associated with commercial heterosexual behavior, including all factors with *p* < 0.10 in bivariate analyses. 624(78.49%) who heard about HIV/AIDS were included in the logistic analysis. Adjusted odds ratio (AOR) and 95% confidence intervals were calculated. Factors with AOR greater than one was categorized as risk factors, and less than one was categorized as protective factors.

## Results

### Basic information

Demographic characteristics and migration experiences are showed in Table 1. Four of 795 (0.50%) respondents admitted that they had used illicit drugs but not through injection. They only used Opium, reported none high-risk sexual behavior, and were tested as HIV negative.

**Table 1:** Demographic characteristics and Migration Experiences of among HIV negative elderly men in Chengdu, China (*n* = 795)

	n(%)		n(%)
Age		Daily entertainment	
50–59	221 (27.8)	Visiting teahouse with friends	512 (64.40)
60–69	375 (47.17)	Playing mahjong	359 (45.16)
70–79	171 (21.51)	Tourism	36 (4.53)
80–87	28 (3.52)	Having party with friends	132 (16.6)
Household residency		Exercises	159 (20.00)
rural	784 (98.6)	Dancing at squares	3 (0.38)
urban	11 (1.4)	Watching TV	452 (56.86)
Latest occupation		Other	146 (18.36)
Farm workers	371 (46.7)	Monthly income	
Laborers	273 (34.3)	No personal income	64 (8.05)
Government employees	28 (3.5)	<1000YUAN	368 (46.29)
Other	123 (15.5)	1000-1999YUAN	191 (24.03)
Education level		2000-2999YUAN	78 (9.81)
Primary school drop outs	350 (44.03)	3000-3999YUAN	52 (6.54)
Primary school	179 (22.52)	4000YUAN and above	42 (5.28)
Junior high school	207 (26.04)	Monthly expense for entertainment	
Senior high school and above	59 (7.42)	< 500YUAN	629 (79.12)
Marital status		500-999YUAN	103 (12.96)
Never married	17 (2.14)	1000-1499YUAN	37 (4.65)
Married	648 (81.51)	1500-1999YUAN	14 (1.76)
Separated	44 (5.53)	2000YUAN and above	12 (1.51)
divorce/widow with fixed sexual partner	16 (2.01)	Migration	
Single (divorce/widow)	70 (8.81)	More than one year ago	382 (48.05)
Living with		Within the past one year	120 (15.09)
Only spouse	306 (38.49)	Never	293 (36.86)
Spouses and other family members (parents or children)	364 (45.79)	Migration site	
Only other family members (parents or children)	41 (5.16)	Other towns in County A	317 (63.15)
Nobody	78 (9.81)	Other subdistricts in the city of Chengdu	165 (32.87)
In facilities	6 (0.75)	Other cities in Sichuan Province	200 (39.84)
		Other provinces	130 (25.95)

### Sexual identity and sexual desire change

Seven hundred ninety-two of 795(99.62%) reported that they are heterosexual, 2(0.25%) as bisexual, and 1 respondent (0.13%) reported that he had never heard of homosexuality. Five hundred forty-two of 795(68.2%) respondents reported that their sexual desire has declined, 19.2% reported no change, 11.9% had no sexual need, and 0.6% reported increasing desire after the age of 50.

### HIV-related health education

Four hundred twenty-seven of 624(68.43%) respondents reported having ever gotten HRHE, mainly through mass media (TV, internet, and radio) (70.49%) and health facilities (CDC, hospitals, and township health centers) (58.31%). Before this investigation, 234 of 427(54.80%) received HRHE from village doctors and health workers from township health center. The details are showed in Table 2.

**Table 2:** Channels of HIV-related Health Education (HRHE) among HIV negative elderly men who had ever gotten HRHE (*n* = 427)

Channel	n (%)	Latest Providers	n (%)
TV, internet, and radio	301 (70.49)	health workers from township health center and village doctors	234 (54.80)
health facilities (CDC, hospitals, and township health centers)	249 (58.31)	public health workers from CDC	111 (26.00)
families, relatives, friends and neighbors	95 (22.25)	civil servant from township governments	67 (15.69)
magazines, newspaper, and books	47 (11.01)	village cadre	49 (11.48)
others	7 (1.64)	others	34 (7.96)
		doctors from other hospitals	23 (5.39)
		grandchild’s teachers	2 (0.47)
		volunteers	1 (0.23)
		pharmacist	0

### HIV-related knowledge level

The HIV-related knowledge awareness rate was 31.41%, and only 20 people (3.21%) scored eight of “China National Eight.” The details are showed in Table 3.

**Table 3:** HIV-related Knowledge awareness among HIV negative elderly men who had heard about HIV/AIDS (n = 624)

item	n (%)
Is it possible to get HIV by sharing a syringe with someone living with HIV/AIDS?	417 (66.83)
Will a person get HIV by transfusion of blood with HIV?	409 (65.54)
Is it possible for a child born to a woman infected with HIV to get HIV?	342 (54.81)
Can a healthy-looking person have HIV?	330 (52.88)
Can having sex with only one faithful, uninfected partner reduce the risk of HIV transmission?	314 (50.32)
Can using condoms reduce the risk of HIV transmission?	264 (42.31)
Can a person get HIV by sharing a meal with someone who is infected?	238 (38.14)
A person can get HIV from mosquito bites.	133 (21.31)
HIV-related Knowledge awareness rate	196 (31.41)
All correct	20 (3.21)

### Sexual attitudes

The majority (41.38%) of 795 respondents reported that they could not accept commercial heterosexual behavior, 29.69% held undecided standpoint, 18.36% said it is understandable as natural human behavior, and only 10.57% reported strong objection. The percentages of corresponding attitudes toward extramarital sexual behaviors were 42.64, 29.56%,12.45 and 14.21%. Moreover, 9 of 795(1.13%) reported that they admired the ones who had extramarital sexual behaviors. In terms of the attitude toward homosexual behavior, 42.26% reported that they do not know what homosexual behavior is, 32.96% strong objection, 17.23% objection, and 7.30% reported it understandable but not acceptable, and 0.25% reported it is understandable and they had homosexual behavior.

### HIV vulnerability and HIV-related stigma

Forty-eight of 624 (7.69%) reported that they were at risk of HIV infection. If their relatives/friends got HIV infected, 29.65% reported that they would be fearful and choose to stay far away, 23.88% would express sympathy but still choose to stay away, 22.60% would show sympathy and want to keep in touch, 12.66% stated they would treat him/her as usual, and only 11.22% would like to care for him/her more.

### Sexual behaviors

Seven hundred eighty-four of 795(98.62%) reported that they had sexual partners in their lifetime. 129 (16.23%) respondents admitted high-risk sexual behaviors, among which the prevalence of commercial heterosexual behavior was 11.07%, extramarital sexual behavior 6.16%, casual sexual behavior 2.89%, and homosexual behavior 0.25%. 20 (2.52%) and 2(0.25%) were involved in two and three types of above high-risk sexual behaviors.

One of the self-identified bisexual respondents reported that he was involved in of the above sex activities throughout his lifetime. Another self-identified bisexual respondent was involved in commercial heterosexual behavior and homosexual behavior.

#### Commercial heterosexual behavior

Eighty-eight of 795(11.07%) respondents admitted they have patronized FCSWs in their lifetime. Their first patronization happened at the age of 33.90 ± 13.43, mainly in other towns in County A (42.53%) where they worked outside of their hometown (83.82%).

These patronizations mostly followed their friends (43.02%) and suggestions from other strangers (40.70%), mainly due to “divorce or separate, purely to solve with physical need” (60.92%), “lonely” (52.87%), and “invited by peers” (43.68%). Generally, they preferred to patronize a different FCSW (62.07%), and 13.79% patronized the same FCSW every time.

Only 16 of 88 (20.00%) reported that they patronized FCSW in the past 1 year, mainly at illegal street side brothels (50.00%) and tea houses (37.50%), at the price of ¥20–49 (3-7USD) (68.75%). None of them used condoms consistently, 68.75% had never used a condom. The main reasons for lack of condom use was “never use condom, don’t know how to use” (43.75%) and “FCSW looks healthy” (31.25%).

### Commercial heterosexual behavior related factors

In bivariate analysis, commercial heterosexual behavior was not related to HIV-related knowledge level, attitude toward extramarital sexual behavior, casual sexual behavior, and homosexual behavior, sexual identity, and sexual desire change(*P > 0.05*). The prevalence of commercial heterosexual behavior among the ones who had ever migrated was 13.55%, higher than among the ones who had never migrated (7.12%)(χ^2^ = 7.734, *P* < 0.01).

Factors associated with commercial heterosexual behavior in bivariate and multivariate analyses are showed in Table 4. The greatest magnitude of a protective factor was undecided (AOR =0.02, 95% CI = 0.01–0.09) and objection (AOR =0.04, 95% CI = 0.01–0.1) attitude toward commercial sex. The greatest risk factors were experiences of work out of town (AOR =2.46,95% CI = 1.02–5.91).

**Table 4:** Association between lifetime commercial heterosexual behavior and related factors among HIV negative elderly men who have heard about HIV/AIDS, bivariate and multivariate analyses (*n* = 624)

	Higher value (%)	UAOR (95%CI)	AOR (95%CI)
Age: ≥60 vs. 50–59	69.39	0.54 (0.34–0.86)	0.41 (0.19–0.91)
Education level			–
Primary school vs. primary school drop outs	21.79	0.64 (0.34–1.21)	
Junior high school vs. primary school drop outs	29.17	1.33 (0.8–2.19)	
Senior high school and above vs. primary school drop outs	8.97	0.26 (0.06–1.12)	
Marital Status			
Married vs. never married	84.29	0.19 (0.07–0.52)	0.04 (0–0.52)
Married but separated vs. never married	5.13	0.41 (0.12–1.43)	0.11 (0.01–2)
Divorced/widowed but have one partner vs. never married	1.76	0.42 (0.09–2.1)	0.22 (0.01–7.1)
Divorced/widowed vs. never married	7.53	0.34 (0.1–1.12)	0.39 (0.02–6.34)
Experiences of work out of town	65.22	2.14 (1.27–3.6)	2.46 (1.02–5.91)
Daily entertainment- exercises	17.95	0.37 (0.17–0.78)	–
Daily entertainment-watching TV	56.09	0.6 (0.38–0.93)	–
Daily entertainment-visiting tea house with friends	67.79	1.76 (1.06–2.91)	–
Monthly expense for entertainment			–
500–999 vs. < 500YUAN	15.87	3.78 (2.25–6.36)	
1000–1499 vs. <500YUAN	4.97	1.29 (0.44–3.78)	
1500–1999 vs. <500YUAN	1.60	0.82 (0.11–6.38)	
2000 and above vs. <500 YUAN	1.60	2.13 (0.45–9.97)	
Receiving HRHE from TV, internet, and radio	70.49	0.33 (0.18–0.61)	0.37 (0.16–0.85)
Self-identified vulnerable to get HIV infected.	7.69	5.53 (2.93–10.45)	–
Attitudes toward commercial sex			
Undecided vs. Accepting it as a natural human behavior	26.92	0.02 (0.01–0.04)	0.02 (0.01–0.09)
Objection vs. Accepting it as a natural human behavior	40.06	0.02 (0.01–0.04)	0.04 (0.01–0.1)
Strongly objection vs. Accepting it as a natural human behavior	11.22	0 (0-)	0 (0-)
Attitudes towards HIV infected family and friends if happens			–
Be fearful and stay far away vs. care for him/her more than before	29.65	1.85 (0.67–5.06)	
Express sympathy but stay far away vs. care for him/her more than before	23.88	3.28 (1.21–8.85)	
Express sympathy and keep in touch vs. care for him/her more than before	22.60	1.66 (0.58–4.75)	
Treat him/her as usual vs. care for him/her more than before	12.66	1.07 (0.31–3.67)	

**Note:** -refers not in the model

## Discussion

Comparing with illicit drug use, high-risk sexual behaviors are more common among elderly men from rural areas in Chengdu. We conducted the investigation in Summer, and missed around 6% [15.09% (within the past 1 year) × 39.84% (other cities in Sichuan Province)] elderly men who migrated outside Chengdu. The adjusted prevalence of commercial heterosexual behavior was 11.22% [(94*11.07%+6*13.55%)/100×100%], and was underestimated by 1.33% [(11.22%-11.07%)/(11.22%)×100%].

It is understandable that single respondents were more likely to be involved in commercial heterosexual behavior. Similar with other elderly men in the world [14], most of the respondents’ sexual desire declined after the age of 50. The older, the less the respondents reported visiting FCSW, but visiting FCSW is not related to sexual desire change. This finding is in concord with the fact that visiting FCSW is not only to fulfill physical needs but emotional needs also [25, 41]. People often think that the conservative attitude could be a restriction against commercial heterosexual behavior [42]. In our survey, it is true among the mild objectors who are against this behavior. Surprisingly statistics found no difference in participating numbers of the commercial heterosexual behavior between the following two groups: accepting it as a natural human behavior verses strong objection. Since the 1980s, sexual attitudes in China have changed from procreation within a family context to being an individual’s responsibility as long as there is no foreseen negative impact on the wellbeing of others or the larger society [42]. However, due to convention [42] and stigma [43], Chinese, especially elderly people, do not talk about sex [44], elderly men involving in commercial heterosexual behaviors may be too shy to admit it. It can only become more difficult to access the HIV education for them than in other age groups.

Worldwide, there is huge regional variation of high-risk sexual behaviors [45], mainly determined by structural factors, such as social and economic context [45, 46]. The greatest risk factors for commercial heterosexual behavior in this study were experiences of work out of town. Since the 1980s, rural-to-urban migration have been part of their lives for men from rural areas in China. In this study, more than half of the respondents had rural-to-urban migration experience. They were sexually active migrating alone [47, 48], easy to be involved in high-risk sexual behaviors [27, 31]. Their first visit to FCSW mostly happened during their migration. Visiting FCSW was cheap and easy to get access. No one used condoms consistently, mainly because of lack of skills and knowledge. Condom in China has been used mainly for contraception, meanwhile the main contraception method among married couples were intrauterine device (IUD) for women [49]. When they were young, respondents did not take major responsibility for contraception, neither did they have too much chances to learn how to use condom, someone even never used condom in their lifetime. Moreover, carrying condom in brothels is taken as an evidence for visiting FCSW in China where sex work is illegal [49], which hold elderly men from initiating condom use when visiting FCSW.

In this study, receiving HRHE through mass media,which cover all groups of population [39] and has been part of the systematic HIV prevention [5], helped elderly men avoid visiting FCSW. Health facilities have played an important role in improving elderly men’s HIV knowledge, but been far from success. In terms of “90–90-90 targets”, even though it is not impossible to achieve the goals of 90% of HIV diagnosed population receiving sustained ART, as well as 90% of those receiving ART be viral suppressed by 2020, it is very hard to achieve 90% of diagnosis rate. Not only because free HIV testing is a voluntary action in China [5], but also one of five elderly men have not even heard the term of HIV/AIDS, let alone utilizing the free test. Current HIV prevention strategies in County A succeed in reducing blood transmission route, and most elderly men are aware of blood transmission route and none use illicit drugs through injections. However, HIV related knowledge level among elderly men is very low (31.41%). There are misunderstandings about transmission routes and stigma toward PLHIV which probably hold them back from utilizing the free test [43]. When they were detected as HIV positive, it is highly possible that they had been infected for a long time. During their undetected HIV status, they would infect their sexual partners unintentionally. FCSW infected by elderly men will also infect other age group men [25, 31–33, 50]. That is why elderly men have been listed as key groups in HIV prevention in 2017 [19].

### Limitations

The current study should be noted including a cross-sectional study design and self-report behavior information. Face-to-face interviews may heighten socially desirable responses such as conservative attitudes toward high-risk behaviors, and low report of high-risk sexual behaviors. In order to confront the problems, our interviewers were well trained, interviews were conducted in separate room, and local slangs were used.

Migration experience is a risk factor for high-risk sexual behavior. At the time of investigation, we missed around 6% elderly men who migrated outside Chengdu. Fortunately, the prevalence of commercial heterosexual behavior in this study was only underestimated by 1.33%.

Due to limited sample size for bisexual respondents, we cannot draw the conclusions that bisexual elderly men were more likely to be involved in high-risk sexual behaviors than heterosexual ones, but the fact that both two self-identified bisexual respondents were involved in commercial heterosexual behaviors gave a clue for further study.

## Conclusions

Comparing with illicit drug use, high-risk sexual behaviors are more common among elderly men from rural areas in Chengdu. HIV related knowledge level among elderly men is very low (31.41%). Receiving HRHE from mass media and undecided and objection attitude toward commercial sex prevent elderly from being involving in commercial heterosexual behavior.

According to the results, health intervention strategies for elderly men could be the following: Health facilities continue to conduct systematic interventions, paying more attention to 50–59 years old group. Sex and condom use need to be talked in public. Working with mass media, health facilities give elderly men health education not only focusing on transmission route of HIV/AIDS, but also on knowledge and skills of condom use.

## Data Availability

The datasets analyzed during the current study are available from the corresponding author on reasonable request.

## Abbreviations

ART: Antiretroviral therapy
HIV/AIDS: Human immunodeficiency virus/Acquired Immune Deficiency Syndrome
UNAIDS: The Joint United Nations Programme on HIV/AIDS
PLHIV: People living with HIV
FCSW: Female commercial sex workers
MSM: Men who have sex with men
CRIMS: the web-based National HIV/AIDS Comprehensive Response Information Management System
CDC: Center for disease prevention and control
ELISA: Enzyme-linked immunosorbent assay
KAP: Knowledge-attitude-practice
HRHE: HIV-related health education
UNGASS: United Nations General Assembly Special Session on HIV/AIDS
AOR: Adjusted odds ratio
IUD: Intrauterine device

## References

[1] Wang H, Wolock TM, Carter A, Nguyen G, Kyu HH, Gakidou E, Hay SI, Mills EJ, Trickey A, Msemburi W, Coates MM, Mooney MD, Fraser MS, Sligar A, Salomon J, Larson HJ, Friedman J, Abajobir AA, Abate KH, Abbas KM, Razek MMAE, Abd-Allah F, Abdulle AM, Abera SF, Abubakar I, Abu-Raddad LJ, Abu-Rmeileh NME, Abyu GY, Adebiyi AO, Adedeji IA, Adelekan AL, Adofo K, Adou AK, Ajala ON, Akinyemiju TF, Akseer N, Lami FHA, al-Aly Z, Alam K, Alam NKM, Alasfoor D, Aldhahri SFS, Aldridge RW, Alegretti MA, Aleman AV, Alemu ZA, Alfonso-Cristancho R, Ali R, Alkerwi A', Alla F, Mohammad R, al-Raddadi S, Alsharif U, Alvarez E, Alvis-Guzman N, Amare AT, Amberbir A, Amegah AK, Ammar W, Amrock SM, Antonio CAT, Anwari P, Ärnlöv J, Artaman A, Asayesh H, Asghar RJ, Assadi R, Atique S, Atkins LS, Avokpaho EFGA, Awasthi A, Quintanilla BPA, Bacha U, Badawi A, Barac A, Bärnighausen T, Basu A, Bayou TA, Bayou YT, Bazargan-Hejazi S, Beardsley J, Bedi N, Bennett DA, Bensenor IM, Betsu BD, Beyene AS, Bhatia E, Bhutta ZA, Biadgilign S, Bikbov B, Birlik SM, Bisanzio D, Brainin M, Brazinova A, Breitborde NJK, Brown A, Burch M, Butt ZA, Campuzano JC, Cárdenas R, Carrero JJ, Castañeda-Orjuela CA, Rivas JC, Catalá-López F, Chang HY, Chang JC, Chavan L, Chen W, Chiang PPC, Chibalabala M, Chisumpa VH, Choi JYJ, Christopher DJ, Ciobanu LG, Cooper C, Dahiru T, Damtew SA, Dandona L, Dandona R, das Neves J, de Jager P, de Leo D, Degenhardt L, Dellavalle RP, Deribe K, Deribew A, Des Jarlais DC, Dharmaratne SD, Ding EL, Doshi PP, Doyle KE, Driscoll TR, Dubey M, Elshrek YM, Elyazar I, Endries AY, Ermakov SP, Eshrati B, Esteghamati A, Faghmous IDA, Farinha CSS, Faro A, Farvid MS, Farzadfar F, Fereshtehnejad SM, Fernandes JC, Fischer F, Fitchett JRA, Foigt N, Fullman N, Fürst T, Gankpé FG, Gebre T, Gebremedhin AT, Gebru AA, Geleijnse JM, Gessner BD, Gething PW, Ghiwot TT, Giroud M, Gishu MD, Glaser E, Goenka S, Goodridge A, Gopalani SV, Goto A, Gugnani HC, Guimaraes MDC, Gupta R, Gupta R, Gupta V, Haagsma J, Hafezi-Nejad N, Hagan H, Hailu GB, Hamadeh RR, Hamidi S, Hammami M, Hankey GJ, Hao Y, Harb HL, Harikrishnan S, Haro JM, Harun KM, Havmoeller R, Hedayati MT, Heredia-Pi IB, Hoek HW, Horino M, Horita N, Hosgood HD, Hoy DG, Hsairi M, Hu G, Huang H, Huang JJ, Iburg KM, Idrisov BT, Innos K, Iyer VJ, Jacobsen KH, Jahanmehr N, Jakovljevic MB, Javanbakht M, Jayatilleke AU, Jeemon P, Jha V, Jiang G, Jiang Y, Jibat T, Jonas JB, Kabir Z, Kamal R, Kan H, Karch A, Karema CK, Karletsos D, Kasaeian A, Kaul A, Kawakami N, Kayibanda JF, Keiyoro PN, Kemp AH, Kengne AP, Kesavachandran CN, Khader YS, Khalil I, Khan AR, Khan EA, Khang YH, Khubchandani J, Kim YJ, Kinfu Y, Kivipelto M, Kokubo Y, Kosen S, Koul PA, Koyanagi A, Defo BK, Bicer BK, Kulkarni VS, Kumar GA, Lal DK, Lam H, Lam JO, Langan SM, Lansingh VC, Larsson A, Leigh J, Leung R, Li Y, Lim SS, Lipshultz SE, Liu S, Lloyd BK, Logroscino G, Lotufo PA, Lunevicius R, Razek HMAE, Mahdavi M, Mahesh PA, Majdan M, Majeed A, Makhlouf C, Malekzadeh R, Mapoma CC, Marcenes W, Martinez-Raga J, Marzan MB, Masiye F, Mason-Jones AJ, Mayosi BM, McKee M, Meaney PA, Mehndiratta MM, Mekonnen AB, Melaku YA, Memiah P, Memish ZA, Mendoza W, Meretoja A, Meretoja TJ, Mhimbira FA, Miller TR, Mikesell J, Mirarefin M, Mohammad KA, Mohammed S, Mokdad AH, Monasta L, Moradi-Lakeh M, Mori R, Mueller UO, Murimira B, Murthy GVS, Naheed A, Naldi L, Nangia V, Nash D, Nawaz H, Nejjari C, Ngalesoni FN, de Dieu Ngirabega J, Nguyen QL, Nisar MI, Norheim OF, Norman RE, Nyakarahuka L, Ogbo FA, Oh IH, Ojelabi FA, Olusanya BO, Olusanya JO, Opio JN, Oren E, Ota E, Park HY, Park JH, Patil ST, Patten SB, Paul VK, Pearson K, Peprah EK, Pereira DM, Perico N, Pesudovs K, Petzold M, Phillips MR, Pillay JD, Plass D, Polinder S, Pourmalek F, Prokop DM, Qorbani M, Rafay A, Rahimi K, Rahimi-Movaghar V, Rahman M, Rahman MHU, Rahman SU, Rai RK, Rajsic S, Ram U, Rana SM, Rao PV, Remuzzi G, Rojas-Rueda D, Ronfani L, Roshandel G, Roy A, Ruhago GM, Saeedi MY, Sagar R, Saleh MM, Sanabria JR, Santos IS, Sarmiento-Suarez R, Sartorius B, Sawhney M, Schutte AE, Schwebel DC, Seedat S, Sepanlou SG, Servan-Mori EE, Shaikh MA, Sharma R, She J, Sheikhbahaei S, Shen J, Shibuya K, Shin HH, Sigfusdottir ID, Silpakit N, Silva DAS, Silveira DGA, Simard EP, Sindi S, Singh JA, Singh OP, Singh PK, Skirbekk V, Sliwa K, Soneji S, Sorensen RJD, Soriano JB, Soti DO, Sreeramareddy CT, Stathopoulou V, Steel N, Sunguya BF, Swaminathan S, Sykes BL, Tabarés-Seisdedos R, Talongwa RT, Tavakkoli M, Taye B, Tedla BA, Tekle T, Shifa GT, Temesgen AM, Terkawi AS, Tesfay FH, Tessema GA, Thapa K, Thomson AJ, Thorne-Lyman AL, Tobe-Gai R, Topor-Madry R, Towbin JA, Tran BX, Dimbuene ZT, Tsilimparis N, Tura AK, Ukwaja KN, Uneke CJ, Uthman OA, Venketasubramanian N, Vladimirov SK, Vlassov VV, Vollset SE, Wang L, Weiderpass E, Weintraub RG, Werdecker A, Westerman R, Wijeratne T, Wilkinson JD, Wiysonge CS, Wolfe CDA, Won S, Wong JQ, Xu G, Yadav AK, Yakob B, Yalew AZ, Yano Y, Yaseri M, Yebyo HG, Yip P, Yonemoto N, Yoon SJ, Younis MZ, Yu C, Yu S, Zaidi Z, Zaki MES, Zeeb H, Zhang H, Zhao Y, Zodpey S, Zoeckler L, Zuhlke LJ, Lopez AD, Murray CJL (2016). Estimates of global, regional, and national incidence, prevalence, and mortality of HIV, 1980-2015: the global burden of disease study 2015. Lancet Hiv.

[2] Lancet T (2017). The global HIV/AIDS epidemic-progress and challenges. Lancet.

[3] Piot P, Abdool Karim SS, Hecht R, Legido-Quigley H, Buse K, Stover J, Resch S, Ryckman T, Møgedal S, Dybul M, Goosby E, Watts C, Kilonzo N, McManus J, Sidibé M (2015). Defeating AIDS—advancing global health. Lancet.

[4] UNAIDS (2014). 90–90-90-an ambitious treatment target to help end the AIDS epidemic.

[5] Zhang, L., et al., Comprehensive HIV/AIDS Programs in Sichuan*.* HIV/AIDS in China 2019: p. 629–651, DOI: 10.1007/978-981-13-8518-6_33.

[6] Degenhardt L, Mathers BM, Wirtz AL, Wolfe D, Kamarulzaman A, Carrieri MP, Strathdee SA, Malinowska-Sempruch K, Kazatchkine M, Beyrer C (2014). What has been achieved in HIV prevention, treatment and care for people who inject drugs, 2010-2012? A review of the six highest burden countries. Int J Drug Policy.

[7] Nguyen N, Holodniy M (2008). HIV infection in the elderly. Compr Ther.

[8] Negin J, Cumming RG (2010). HIV infection in older adults in sub-Saharan Africa: extrapolating prevalence from existing data. Bull World Health Organ.

[9] Qiao Y-C (2019). Epidemiological analyses of regional and age differences of HIV/AIDS prevalence in China, 2004–2016. Int J Infect Dis.

[10] Liu H, Lin X, Xu Y, Chen S, Shi J, Morisky D (2012). Emerging HIV epidemic among older adults in Nanning, China. AIDS Patient Care STDS.

[11] High KP (2012). HIV and aging: state of knowledge and areas of critical need for research. A report to the NIH Office of AIDS Research by the HIV and Aging Working Group. Jaids J Acquired Immune Deficiency Syndromes.

[12] Smit M, Brinkman K, Geerlings S, Smit C, Thyagarajan K, Sighem Av, de Wolf F, Hallett TB, ATHENA observational cohort (2015). Future challenges for clinical care of an ageing population infected with HIV: a modelling study. Lancet Infect Dis.

[13] Leng SX, Margolick JB (2020). Aging, sex, inflammation, frailty, and CMV and HIV infections. Cell Immunol.

[14] Diokno AC, Brown MB, Herzog AR (1990). Sexual function in the elderly. Arch Intern Med.

[15] Zhang L, Chow EPF, Jing J, Zhuang X, Li X, He M, Sun H, Li X, Gorgens M, Wilson D, Wang L, Guo W, Li D, Cui Y, Wang L, Wang N, Wu Z, Wilson DP (2013). HIV prevalence in China: integration of surveillance data and a systematic review. Lancet Infect Dis.

[16] Kang S-C, Hwang S-J, Wong W-W (2011). Characteristics of human immunodeficiency virus infections among the elderly in Taiwan: a nationwide study. J Chin Med Assoc.

[17] Jia ZW, et al. Tracking the Evolution of HIV/AIDS in China from 1989–2009 to Inform Future Prevention and Control Efforts. PLoS One. 2011;6(10). 10.1371/journal.pone.0025671. 10.1371/journal.pone.0025671 PMC3187780 21998679

[18] Cui Y, Shi CX, Wu Z (2017). Epidemiology of HIV/AIDS in China: recent trends. Global Health J.

[19] Council, O.o.C.S (2017). China's 13th Five-year Action plan for AIDS prevention and control.

[20] Tucker JD, Henderson GE, Wang TF, Huang YY, Parish W, Pan SM, Chen XS, Cohen MS (2005). Surplus men, sex work, and the spread of HIV in China. Aids.

[21] Thakor HG, Kosambiya JK, Desai VK (2010). Knowledge and practices related to sexually transmitted infections and HIV among sex workers in an urban area of Gujarat, India. J Indian Med Assoc.

[22] Siddiqui AUR (2011). Condom use during commercial sex among clients of Hijra sex workers in Karachi, Pakistan (cross-sectional study). BMJ Open.

[23] Reilly KH, Wang J, Zhu Z, Li S, Yang T, Ding G, Qian HZ, Kissinger P, Wang N (2012). HIV and associated risk factors among male clients of female sex workers in a Chinese border region. Sex Transm Dis.

[24] Zhang C, Li X, Su S, Zhang L, Zhou Y, Shen Z, Tang Z (2014). Prevalence of HIV, syphilis, and HCV infection and associated risk factors among male clients of low-paying female sex workers in a rural county of Guangxi, China: a cross-sectional study. Sex Transm Infect.

[25] Yang Y, Yang C, Latkin CA, Luan R, Nelson KE (2016). Condom Use During Commercial Sex Among Male Clients of Female Sex Workers in Sichuan China: A Social Cognitive Theory Analysis. AIDS Behav.

[26] Lau JTF, Wang M, Wong HN, Tsui HY, Jia M, Cheng F, Zhang Y, Su X, Wang N (2008). Prevalence of bisexual behaviors among men who have sex with men (MSM) in China and associations between condom use in MSM and heterosexual behaviors. Sex Transm Dis.

[27] Song Y (2011). HIV-testing behavior among young migrant men who have sex with men (MSM) in Beijing, China. Aids Care.

[28] Liao M, Kang D, Jiang B, Tao X, Qian Y, Wang T, Bi Z, Xiao Y, Li C, Wu P, Vermund SH, Jia Y (2011). Bisexual behavior and infection with HIV and syphilis among men who have sex with men along the east coast of China. AIDS Patient Care STDS.

[29] Huang L (2014). Sociodemographic and sexual behavior characteristics of an online MSM sample in Guangdong, China. AIDS Care.

[30] Chow EPF, Lau JTF, Zhuang X, Zhang X, Wang Y, Zhang L (2014). HIV prevalence trends, risky behaviours, and governmental and community responses to the epidemic among men who have sex with men in China. Biomed Res Int.

[31] Wang B, Li X, Stanton B, Fang X, Lin D, Mao R (2007). HIV-related risk behaviors and history of sexually transmitted diseases among male migrants who patronize commercial sex in China. Sex Transm Dis.

[32] Fosados R, Caballero-Hoyos R, Torres-López T, Valente TW (2006). Condom use and migration in a sample of Mexican migrants: potential for HIV/STI transmission. Salud Publica Mex.

[33] Saggurti N (2011). Male migration and risky sexual behavior in rural India: Is the place of origin critical for HIV prevention programs?. BMC Public Health.

[34] Zhang TQ (2019). Awareness of HIV/AIDS and its routes of transmission as well as access to health knowledge among rural residents in Western China: a cross-sectional study. BMC Public Health.

[35] Stall R, Catania J (1994). AIDS Risk Behaviors Among Late Middle-aged and Elderly Americans.

[36] Tuddenham SA, Page KR, Chaulk P, Lobe EB, Ghanem KG (2017). Patients fifty years and older attending two sexually transmitted disease clinics in Baltimore, Maryland. Int J STD AIDS.

[37] Fsya B, et al. Epidemiological and spatiotemporal analyses of HIV/AIDS prevalence among older adults in Sichuan, China between 2008 and 2019: a population-based study - ScienceDirect. Int J Infect Dis. 2021. 10.1016/j.ijid.2021.02.077 33618006

[38] Zhenhua D, Shuangfeng F, Rong L, Xueqing W, Yaying S, Zhijun L, Weihua J, Fang L, Zhen D, Xiaodong W, Yujing Z, Qinying H (2015). Consistently high HIV prevalence among men who have sex with men in Chengdu city from 2009 to 2014. Int J STD AIDS.

[39] Ministry of Industry and Information Technology of the People's Republic of China. Telephone users by province in June 2019. 2019. Available from: https://www.miit.gov.cn/gxsj/tjfx/txy/art/2020/art_b255416f73ec483fb754ac621f19217f.html.

[40] Organization, G.W.H (2007). Monitoring the Declaration of Commitment on HIV / AIDS. Guidelines on construction of core indicators. 2008 reporting.

[41] Pan S, Parish WL, Huang Y (2011). Clients of female sex workers: a population-based survey of China. J Infect Dis.

[42] Zhang K, Beck EJ (1999). Changing sexual attitudes and behaviour in China: implications for the spread of HIV and other sexually transmitted diseases. Aids Care.

[43] Genberg BL, Hlavka Z, Konda KA, Maman S, Chariyalertsak S, Chingono A, Mbwambo J, Modiba P, van Rooyen H, Celentano DD (2009). A comparison of HIV/AIDS-related stigma in four countries: negative attitudes and perceived acts of discrimination towards people living with HIV/AIDS. Soc Sci Med.

[44] Yang Y, Luan RS, Liu P, Wu CL, Zhou Y, Chen W (2012). Casual sex and concurrent sexual partnerships among young people from an Yi community with a high prevalence of HIV in China. Asian J Androl.

[45] Wellings K, Collumbien M, Slaymaker E, Singh S, Hodges Z, Patel D, Bajos N (2007). Sexual behaviour in context: a global perspective. Lancet.

[46] Shannon K, Strathdee SA, Goldenberg SM, Duff P, Mwangi P, Rusakova M, Reza-Paul S, Lau J, Deering K, Pickles MR, Boily MC (2015). Global epidemiology of HIV among female sex workers: influence of structural determinants. Lancet.

[47] Wu XG, Treiman DJ (2004). The household registration system and social stratification in China: 1955-1996. Demography.

[48] Wong DFK, Li CY, Song HX (2007). Rural migrant workers in urban China: living a marginalised life. Int J Soc Welf.

[49] Zou H, Xue H, Wang X, Lu D (2012). Condom use in China: prevalence, policies, issues and barriers. Sex Health.

[50] Yang H, Li X, Stanton B, Liu H, Liu H, Wang N, Fang X, Lin D, Chen X (2005). Heterosexual transmission of HIV in China: a systematic review of behavioral studies in the past two decades. Sex Transm Dis.

